# Transferability of Data Sets between Machine-Learned Interatomic Potential Algorithms

**DOI:** 10.1021/acs.jctc.5c00272

**Published:** 2025-06-05

**Authors:** Samuel P. Niblett, Panagiotis Kourtis, Ioan-Bogdan Magdău, Clare P. Grey, Gábor Csányi

**Affiliations:** † Yusuf Hamied Department of Chemistry, 2152 University of Cambridge, Lensfield Road, Cambridge CB2 1EW, U.K.; ‡ School of Natural and Environmental Science, 5994 Newcastle University, Newcastle upon Tyne NE1 7RU, U.K.; § Engineering Laboratory, 98458 University of Cambridge, Trumpington St and JJ Thomson Ave, Cambridge CB2 1PZ, U.K.

## Abstract

The emergence of Foundational Machine Learning Interatomic Potential (FMLIP) models trained on extensive data sets motivates attempts to transfer data between different ML architectures. Using a common battery electrolyte solvent as a test case, we examine the extent to which training data optimized for one machine-learning method may be reused by a different learning algorithm, aiming to accelerate FMLIP fine-tuning and to reduce the need for costly iterative training. We consider several types of training configurations and compare the benefits they bring to feedforward neural networks (the Deep Potential model) and message-passing networks (MACE). We propose a simple metric to assess model performance and demonstrate that MACE models perform well with even the simplest training sets, whereas simpler architectures require further iterative training to describe the target liquids correctly. We find that configurations designed by human intuition to correct systematic deficiencies of a model often transfer well between algorithms, but that reusing configurations that were generated automatically by one MLIP does not necessarily benefit a different algorithm. We also compare the performance of these bespoke models against two pretrained FMLIPs, demonstrating that system-specific training data are usually necessary for realistic models. Finally, we examine how training data sets affect a model’s ability to generalize to unseen molecules, finding that model stability is conserved for small changes in molecule shape but not changes in functional chemistry. Our results provide insight into how training set properties affect the behavior of an MLIP and principles to enhance training sets for molecular liquid models with minimal computational effort. These approaches may be used in tandem with FMLIPs to dramatically accelerate the rate at which new chemical systems can be simulated.

## Graphical abstract

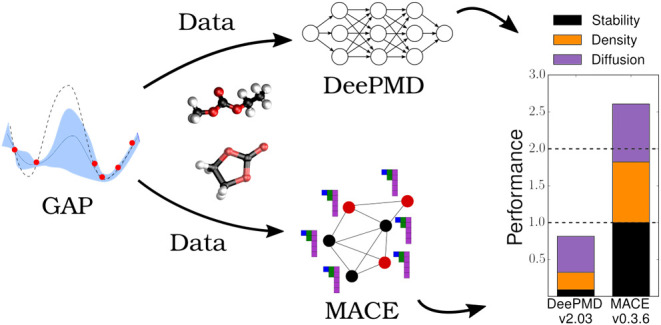

## 1 Introduction

Recent years have seen an explosion in the field of atomistic simulation for materials and complex liquids, driven both by the burgeoning importance of electrochemical^1–5^ and nanostructured devices^6,7^ to enhance sustainable technology, and by the emergence of machine-learning interaction potentials (MLIPs) as a simulation method that approaches quantitative accuracy relative to experiments. MLIPs represent an effective compromise between the accuracy of *ab initio* simulations and the large system sizes that can be studied using classical molecular dynamics (MD), facilitating simulations of complex molecular systems that are directly comparable to experiments.^8–10^ A particularly exciting achievement is the emergence of models that can describe bond-breaking chemical reactions on time and length scales sufficient to simulate complex environments such as electrolytes and solid/liquid interfaces.^11–14^

### 1.1 Principles of MLIPs

MLIPs predict the energy and atomic forces associated with given molecular arrangements using supervised learning, offering lower computational costs than density functional theory (DFT) and scaling well to large systems.^15^ Typically one trains an MLIP to predict the DFT energies for small simulation sizes and then applies it to a much larger problem. Early MLIP models gave excellent agreement with reference calculations for configurations of the system that were similar to their training data, but were poor at extrapolating their energy and force predictions to describe unseen atomic environments, and therefore often unstable in molecular dynamics (MD) simulations.^11^ This issue is particularly acute for molecular liquids, which have intramolecular degrees of freedom and require large simulation cells; hence, their trajectories explore large configuration spaces and are very likely to encounter unseen environments. Once a trajectory leaves the well-trained region of the underlying model, inaccurate energies and forces may prevent it from returning, often leading to catastrophic failure of the simulation. Even without such a failure, regions of the potential energy surface with high prediction errors can dramatically alter the configurational probability distribution for the model,^16^ leading to erroneous predictions of thermodynamic and kinetic properties.

To reduce the requirement for extrapolative prediction and thus reduce model errors, we theoretically desire a training set that represents the target system’s entire thermodynamically accessible configuration space, equivalent to a full ergodic sample. However, obtaining such a sample is impractical for any situation where an MLIP is desired. Instead, one typically constructs a small initial training set that aims to describe the high-probability regions of configuration space (usually from a short *ab initio* simulation or using a classical force field to generate configurations) and then refines it to eliminate “holes”. Holes are regions of configuration space where poor representation in the training set produces high prediction errors.^11^ Training data for model refinement can be generated by hand. For example, in ref 17, it was shown that capturing the correct density of a molecular liquid with an MLIP is challenging, and training configurations with nonequilibrium densities were deliberately created to help correct this issue. The consequences and utility of that approach are discussed further below. A more common method, subsequently referred to as “iterative training”, is to use the MLIP itself to generate new configurations through MD or Monte Carlo simulation and incorporate some of these into the training set, thus improving the MLIP’s performance in precisely the regions of configuration space that will be explored in later production simulations. Different approaches may be used to select the subset of configurations that are incorporated,^18^ for example selecting configurations with particularly large errors relative to reference DFT calculations, or configurations with anomalous values of a collective quantity (e.g., density). One particularly important method uses an uncertainty quantification metric that does not involve explicit calculation of reference energies (for example, the variance of predicted energies across a set of equivalent models called a committee)^11,19^ and selects configurations with high uncertainty to use in model refinement. This special case of iterative training is often called “active learning” and will be employed in Section 3.2. Active learning procedures can be very effective but also computationally expensive and difficult to converge.

### 1.2 Motivation: Data Reuse in Bespoke and Fine-Tuned MLIPs

Recently, the foundational model approach has disrupted the MLIP field: the advent of efficient and smooth ML algorithms (particularly those based on equivariant graph neural networks)^20–23^ as well as large, diverse, public data sets, has made it possible to train models that describe an extensive chemical space.^24–29^ Many of these foundational models appear to have far fewer holes in their well-trained configuration spaces, and thus provide robust molecular dynamics simulations for many different applications.^27^ However, this generality means that they often do not provide *ab initio* accuracy in computing thermodynamic or kinetic properties for any specific system; therefore, additional training data specific to the target process must be added to use them in a predictive fashion. This process helps to specialize (or fine-tune) the model to describe a particular system or process correctly.^27,30,31^ Methods of producing training data for fine-tuning are currently being explored, and a natural question arises whether pre-existing MLIP training sets can be reused for this purpose. Moreover, understanding when and whether it is useful to combine different training sets is an important component of developing a reliable fine-tuning strategy. Even for bespoke MLIPs not based on foundational models, combining and reusing training data sets is an appealing approach to minimize the amount of iterative training required to achieve the desired model accuracy. As the MLIP field expands, researchers increasingly find that the system they are interested in has already been studied but often with a different ML algorithm or set of hyperparameters than they intend to use. Thanks to many funders’ Open Data policies, the data sets for these models are often publicly available, so training on the existing data set might offer a quick route to a usable MLIP (or at least reduce the number of iterative training generations required).

However, it is not clear whether data that were tailored to improve the performance of one MLIP algorithm are useful or relevant to another. We expect that MLIPs with different functional forms that are trained on the same data set will give very different extrapolative predictions in the same regions of configuration space. Therefore, the configurations generated by iterative training on two such models will also be very different, and training on the optimized data set of one MLIP may not prevent a model of a different class from exploring unphysical configurations. Under this hypothesis, configurations obtained by active learning procedures should provide little benefit when they are transferred between MLIP architectures.

### 1.3 Objectives

This paper aims to explore the opportunities and limitations of data reuse by examining how much the training configurations that improve one MLIP architecture can help to improve model accuracy for a different type of MLIP. Our analysis will include both iteratively generated configurations (harvested from the dynamics of the original MLIP) and nonequilibrium manually generated configurations that are designed to correct predicted properties of a target system. We use bespoke MLIPs of molecular liquid mixtures as an example and consider several classes of MLIP. We base our investigation around three questions:

1. How should the performance of MLIPs be quantified/compared, and how does training data affect different aspects of performance?
2. What types of training configuration provide most benefit to an MLIP, and can these configurations be transferred between different algorithms?
3. Which types of training configuration provide most benefit to the generalization ability of an MLIP (i.e., its ability to describe chemical systems not included in the training set)?

### 1.4 MLIP Models of Battery Electrolytes

To investigate the three research questions defined in Section 1, we train MLIPs for a liquid mixture of small organic molecules: ethylene carbonate (EC) and ethyl methyl carbonate (EMC). The structures of these molecules, and others referred to subsequently, are shown in Figure 1. This mixture is representative of the typical challenges facing any model for molecular liquids: large molecular dipoles, a combination of stiff and soft intramolecular degrees of freedom, and a variety of local compositions that individual molecules may sample (ranging from pure EC to pure EMC in a given volume of liquid).

**Figure 1:**
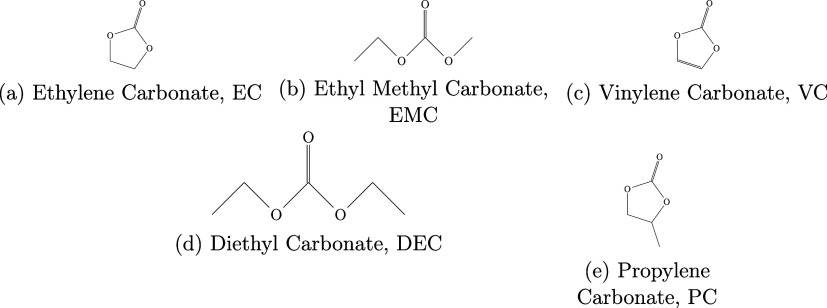
Structures of the molecules used in this work.

More importantly, these mixtures are commonly used as solvents for lithium-ion battery electrolytes^32^ and their reactive decomposition is a key step in degradation mechanisms that limit battery lifetime.^33^ Simulating the physical properties (and ultimately the reactivity) of these liquids is therefore an important goal for atomistic modeling, that has already received significant attention from the MLIP community.^17,34,35^

For example, teams based at Schrödinger^34^ and ByteDance^35^ have both produced MLIPs that reproduce multiple liquid properties for several combinations of organic electrolyte solvents and lithium salt. Both groups trained their models on gas-phase molecular clusters extracted from liquid-state configurations. Also, both used a charge-equilibration scheme to predict the atomic partial charges and hence calculate electrostatic interactions (including the long-ranged component) using Coulomb’s law rather than a short-ranged machine-learned potential. This step is likely to be important in any model that is applied to heterogeneous systems.^36^ For MLIPs that are only used to study bulk liquids (such as our models discussed below), it is sufficient to use local MLIP predictions of electrostatic energies.^37^

These prior studies differ substantially in their MLIP architecture: ref 34 employed a feedforward neural-network model with Behler-Parrinello symmetry functions,^38^ while ref 35 used a novel graph-equivariant-transformer architecture and an ensemble knowledge distillation scheme (i.e., predicted forces were averaged across a committee of equivalent models). An interesting innovation in the latter case was modifying the MLIP forces to align the predicted liquid density with the experimental data. This approach offers to escape the dependence on accuracy of reference calculation (e.g., choice of DFT functional) that typically hinders MLIP studies, but at the cost of potentially introducing additional noise and bias into the predicted forces.

The models we test in the subsequent sections follow a rather different training procedure. We use MLIPs alone to predict energies and forces (without explicit electrostatics), and we train on configurations of liquids under periodic boundary conditions with small simulation cells, rather than on gas-phase clusters. Comparing the relative merits of these two training strategies is an interesting question, but is not the focus of the present article. Instead, we focus on the effect of the training set makeup on the performance of models with different MLIP architectures.

Many different electrolyte formulations have been studied experimentally, including a wide range of compositions. For simplicity, most of our work that follows will aim to reproduce properties for a single composition: the 1:2 molar mixture of EC and EMC corresponding to a 3:7 weight ratio at room temperature, which is a very common battery electrolyte solvent.^39^ We note for clarity that it is common in the experimental literature to express electrolyte compositions as weight or volume ratios; we will use molar compositions unless otherwise stated.

Experimental battery materials research often involves comparing properties of multiple cells with distinct but related chemistries, e.g., varying the electrolyte composition. To emulate and support this workflow, it is desirable to have a single MLIP capable of describing a range of electrolyte molecules, ideally without needing expensive, explicit training for each. It is therefore instructive to consider how our models generalize to molecules not included in the training set and whether some types of training configuration confer more benefit for this task. In Section 4.2, we consider how MLIP models trained to describe EC/EMC mixtures perform when tasked with simulating EC/DEC, VC/EMC, and PC, all of which are also common electrolyte molecules. Their structures are given in Figure 1.

Generalizability of models to study multiple chemical systems has been studied previously,^35,40–42^ and various model types have been shown to be effective. Moreover, Goodwin et al. investigated how training set composition affects generalization between different molar ratios of a multicomponent ionic liquid, making several valuable observations to which we will refer subsequently.^41^ However, to our knowledge, the present article is the first to explore the effect of training data and data reuse on generalizability to different molecular structures.

### 1.5 Classes of MLIP

A great many MLIP protocols have emerged in recent years, using various machine-learning algorithms.^16^ Their philosophies and performance vary considerably and are difficult to assess generally due to the vast number of methods used and because different types of models have different advantages and problems to which they are best suited. This diversity makes the ability to recycle data from one protocol to another even more important: even where a well-trained model for a particular problem exists, other groups may wish to use the same data with a different protocol for reasons of efficiency, expertise, or hardware compatibility.

Our study considers three MLIP methods representing quite different classes of learning algorithm. These are

1. GAP,^43^ a Gaussian Process regression model that represents atomic configurations using the three-body turbo SOAP (Smooth Overlap of Atomic Positions) descriptors.^44^
2. DeePMD,^45^ an end-to-end feedforward neural network. The descriptor functions are generated by an embedding neural network that combines two-body interatomic vectors to produce a many-body descriptor. Regression of these descriptors to the atomic energies and forces is performed by a separate multilayer perceptron.
3. MACE,^23^ an equivariant message-passing neural network that combines radial and angular features across multiple interaction steps to produce many-body descriptors. Multiple message-passing iterations provide not only a higher body-order of descriptor but also a wider receptive field for a given radial cutoff. Regression to energies is performed by a multilayer perceptron.

Features of these architectures will be discussed where they become relevant in the text. The main point to note here is that these models use three different types of symmetrized representation (turbo SOAP, neural-network embeddings of radial and angular information, and many-body atomic clusters produced by message-passing) and two very different regression algorithms (Gaussian process regression and artificial neural networks). Our results are not restricted to a particular class of models, suggesting that the conclusions are somewhat general. However, the breadth of MLIP architectures available means that some may behave very differently from the results we present below. Moreover, the extensive numerical tests that we have performed mean that we have only been able to study data transfer in one direction (from GAP to Deepmd and MACE). The differences in behavior between models trained on these three architectures are striking enough to make confident predictions about data transfer between different MLIPs that have similar properties.

The structure of the paper is as follows: Section 2 introduces our approach to training MLIPs (further details in Supporting Information), to investigating data transferability and assessing MLIP performance. In Section 3.1, we compare how DeePMD and MACE models perform on transferred data sets, and explore the effect of transferred data on active learning efficiency in Sections 3.2 and 3.3. Section 4 contains two brief preliminary studies that compare our bespoke models against two FMLIPs and consider how training data affects chemical generalization. These are not exhaustive investigations but are intended to illustrate the potential benefits of carefully reusing training data. We conclude by summarizing our results and offering an outlook on the scope for data reuse in MLIP simulations of battery and other materials.

## 2 Methodology

MLIPs were trained using DeePMD-kit version 2.0.3 ^45,46^ and MACE version 0.3.6.^23,47^ Further improvements have been made to both architectures and to their software implementations in subsequent versions, but the fundamental properties of each approach, particularly the fitting architectures and the general nature of the descriptor functions, are unchanged. We chose reasonable hyperparameters for each MLIP algorithm based on our group’s earlier work (the values used and other model details are presented in Section S1) but were unable to perform a comprehensive hyperparameter optimization due to the number of different models and systems being tested.

Details of time and computing resources required for training and inference are provided in Section S2. We note here that training costs were comparable for DeePMD and MACE (within a factor of 2 using our typical parameters, although the DeePMD training cost could be reduced by a factor of 100 if we accepted greatly increased walltimes for training). Simulation costs with MACE were 3–4 times higher than those with DeePMD at the system size studied. We refer the reader to ref 48 for further details regarding MACE computational costs and how these may be reduced.

This study should not be construed as a definitive comparison of the best-case performance or efficiency for each software package but rather as an analysis of how a typical user’s models might respond to varying data types.

### 2.1 Transferability of Different Training Configuration Types

The core idea of this work is an ablation study to compare the performance of MLIPs trained on different subsets of a complex optimized training set. This procedure helps us to understand which types of configuration are the most transferable between architectures and what properties of the new model they affect. The training set that we use for this purpose is that reported in ref 17, which was developed to train a GAP MLIP for the EC/EMC mixture at various compositions. This data set was constructed without using *ab initio* MD simulations, significantly reducing the initial cost to produce it, and contains around 1000 configurations (quite a small training set by the standard of molecular MLIPs). By reducing this set further we deliberately enter an extremely low-data regime to discover which parts of the data set are needed to capture each system property correctly.

Throughout, reference energies were calculated using the plane-wave implementation of CASTEP^49^ using the PBE exchange-correlation functional with D2 dispersion correction (G06 keyword),^50^ a plane-wave energy cutoff of 800 eV, and a Monkhorst–Pack 1 × 1 × 1 *k*-point grid. Standard CASTEP ultrasoft pseudopotentials were used to model the core electrons. The GAP training set contained the following types of molecular configuration:

1. 200 liquid configurations obtained from molecular dynamics simulations using the OPLS-AA force field^51^ at a constant composition of 4 EC molecules and 8 EMC molecules (160 atoms). 120 configurations were taken from NPT simulations at various elevated temperatures and pressures, and 80 from NVT simulations at 300–400 K. The NVT set covers a range of state points, including densities as low as half the equilibrium value.
2. Single isolated molecules of EC and EMC in a variety of near-equilibrium configurations (200 configurations of EC and 400 of EMC). DFT energies and forces were evaluated in a periodic cell with dimensions large enough to avoid finite-size effects.
3. Volume scan data: configurations obtained by taking frames from a GAP-MD trajectory and isotropically expanding or contracting them to obtain varying density, as discussed in ref 17. Molecules are kept rigid so that only the intermolecular separation is varied. The original data set contained scans at multiple compositions, but to focus on the effect of the volume-scan method, we included only 3 scans (20 configurations) that have the target composition of 4 EC and 8 EMC molecules.
4. 150 configurations obtained by 12 generations of iterative training using the GAP potential. At each generation, 5–20 configurations with anomalous densities were selected and added to the training set. These configurations span a range of densities and compositions, but all contain a total of 12 molecules.

To assess the transferability of each type of configuration, we train a series of MLIPs using DeePMD and MACE whose initial training data are taken from different subsets of the GAP data set. Specifically, we trained models using only data set 1 (“OPLS only”), sets 1 and 2 (“OPLS + SM”), sets 1 and 3 (“OPLS + VS”), and all four sets combined (“Full”). Note that only the “full” set samples all possible EC/EMC compositions, and only this set contains configurations that were obtained by iterative training of the GAP model. We compare the performance of these MLIPs using several properties, including a combined performance metric introduced in the following section.

### 2.2 Assessing Model Performance Using Liquid-State Properties

MLIPs are often assessed using prediction errors for energies and forces relative to their reference values, usually DFT calculations. These errors are averaged across fixed sets of testing configurations, which are usually held back from the original training set. This approach quantifies performance within the restricted volume of configuration space defined by the test set and thus provides a controlled comparison of quality for different MLIPs for the same chemical system. However, errors computed this way do not necessarily correlate with a model’s ability to reproduce properties of the target material.^18^ A more relevant but more expensive test of model quality is to validate predicted properties against reference values where these are available. For a liquid, we propose that three fundamental properties are the most important: trajectory stability, liquid density, and self-diffusivity.

Computing equilibrium properties typically requires at least nanoseconds of MD simulation, so the ability to propagate long trajectories without encountering significant unphysical behavior is the most fundamental property required of an MLIP. We diagnose this “trajectory stability” by measuring the time period, *t*_stab_, that may be simulated without encountering dramatic changes in molecular geometry or liquid structure (e.g., evaporation) that would not likely occur in equivalent simulations using the reference DFT method. We define *t*_stab,NVT_ as the simulated time elapsed before a canonical MD trajectory undergoes bond-breaking: one or more of the initial chemical bonds exceeds its average length by 0.5 Å. An equivalent quantity for isothermal–isobaric trajectories, *t*_stab,NPT_, measures the time until either a bond breaks or the liquid density leaves the range [0.2,2.0] g/cm^3^. Meeting either of these conditions indicates a catastrophic failure of the trajectory and we have observed that simulations rarely, if ever, recover from such a position. In order to test stability very strictly, we evaluate trajectories at 500 K so that high-energy configurations are comparatively easy to access. An MLIP that is stable at this temperature must have achieved small prediction errors for a large volume of the liquid configuration space, in order to disfavor unphysical configurations.

Liquid density and self-diffusivity are natural measures of a liquid’s thermodynamic and dynamic properties. They are simple to compute from a moderately long MD trajectory, strongly correlated with more complex liquid properties including conductivity, and sensitive to both low- and high-energy configurations of the liquid. The ability of MLIP to reproduce reference values for these properties is therefore an appropriate measure of its accuracy.

As mentioned previously, our MLIPs are trained against DFT data with the PBE exchange-correlation functional and the D2 dispersion correction. Therefore, a well-performing MLIP model should reproduce the density and diffusivity of that reference method rather than experimentally correct values. For example, the 500 K temperature at which we have run our tests is likely above the experimental boiling point of the 1:2 EC/EMC mixture being studied. However, PBE-D2 simulations predict a stable liquid under these conditions, so we consider this behavior to be “correct” for the purposes of assessing model performance.

Measuring reliable density distributions and diffusivities at the PBE-D2 level was impractical due to the high computational cost of *ab initio* MD. Instead, we compare the predictions of our MLIPs against those of the final GAP model in ref 17 (labeled as Gen16/DTS in that paper), which we refer to subsequently as the “Target” model. This model was previously validated carefully against both AIMD and experimental data to confirm that it gives reasonable predictions for the liquid density. However, DFT data were not available to validate the Target model’s diffusivity. This point will be discussed further in Section 3.1.

Taking all these considerations together, we suggest the following simple metric, *Q*, to evaluate the model performance. *Q* comprises terms for each of: trajectory stability, density, and diffusivity, assigning a score on [0,1] for each. A score of 1 indicates close agreement with the target GAP model; 0 indicates complete prediction failure.

1 $$Q=\langle Q_{\mathrm{stab}}+Q_{\rho }+Q_{D}\rangle_{\mathrm{comm}}$$

where ⟨···⟩_comm_ indicates a committee average: for each MLIP architecture and training set, we train 5 equivalent models, differing only by random seed used to initialize training. *Q* is an average across the predictions of these 5 models. The individual terms are defined as follows.

2 $$Q_{\mathrm{stab}}=\min\left(1,\frac{t_{\mathrm{stab},\mathrm{NPT}}}{1\mathrm{ns}}\right)$$

We find that trajectories that are stable for 1 ns usually remain stable for much longer times, so we assign the maximum score of 1 to trajectories satisfying this condition. Trajectories that fail sooner receive a proportionately smaller *Q*_stab_.

$$Q_{\rho }=\{\begin{array}{ll} 1-\tan h\left(\mathrm{KL}\left(P_{\mathrm{ML}}\left(\rho \right)∥P_{\mathrm{ref},\mathrm{av}}\left(\rho \right)\right)\right) & \mathrm{if}\;t_{\mathrm{stab},\mathrm{NPT}}>0.1\;\mathrm{ns}\\ 0 & \mathrm{otherwise} \end{array}$$ 3

which compares the predicted density of the MLIP being tested against that of the GAP reference model. The Kullback–Leibler divergence $\mathrm{KL}\left(P∥Q\right)=\sum_{x}P\left(x\right)\log\frac{P\left(x\right)}{Q\left(x\right)}$ is used to quantify the dissimilarity of two probability distributions. *P*_ML_(ρ) and *P*_ref_(ρ) indicate distributions of the instantaneous density predicted by the test and reference models. This distribution is sensitive to the model’s predictive accuracy in both high- and low-probability regions of phase space, and its width determines the isothermal compressibility of the liquid. Our definition of *Q*_ρ_ is therefore an informative and stringent test of important thermodynamic properties of a given MLIP. We estimate *P*_ML_(ρ) with a discretized sample from an isothermal–isobaric trajectory, sampling from the time interval [0,*t*_stab_] to ensure that only physically reasonable configurations contribute to the sample.

For an accurate MLIP with *P*_ML_(ρ) ≈ *P*_ref_(ρ), we would have KL = 0 and *Q*_ρ_ = 1. The tanh function acts as a switch: as the divergence increases, the second term becomes more negative, and *Q*_ρ_ tends to 0. One could scale the argument of the switch to set the range of KL values that give non-negligible *Q*_ρ_, but our tests found that unit scaling gives an intuitive measure of dissimilarity in distributions. *Q*_ρ_ = 0.1 corresponds roughly to the case in which two normal distributions of equal width have their means offset by one standard deviation. Finally,

4 $$Q_{D}=\{\begin{array}{ll} 1-\tanh\left(\frac{\left|D_{\mathrm{ML}}-D_{\mathrm{ref}}\right|}{D_{\mathrm{ref}}}\right) & \mathrm{if}\;t_{\mathrm{stab},\mathrm{NVT}}>0.1\;\mathrm{ns}\\ 0 & \mathrm{otherwise} \end{array}$$

quantifies the deviation of the center-of-mass diffusivity predicted by the MLIP, *D*_ML_, from its reference value *D*_ref_. As before, the tanh function is used to assign *Q*_D_ = 1 when these quantities are equal and 0 when they are very different. Each diffusion coefficient is evaluated using the Einstein formula *D*_μ_ = < |**r**_μ_(*t*) – **r**_μ_(0)|^2^ > /6*t*, where **r**_μ_ is the center-of-mass position for a particular molecule of type μ. The exact approach used is described in the caption of Figure 2. When computing *Q*_D_, we average the diffusivities of the two molecular components (EC and EMC), weighted by their mole fractions, and thus obtain a single measure of diffusivity to compare. The EC and EMC diffusivities are usually similar in any case. All quantities contributing to *Q*_D_ are evaluated on the interval [0,*t*_stab,NVT_] using a canonical trajectory at the equilibrium density of the GAP reference system: 0.92 g/cm^3^.^17^

**Figure 2:**
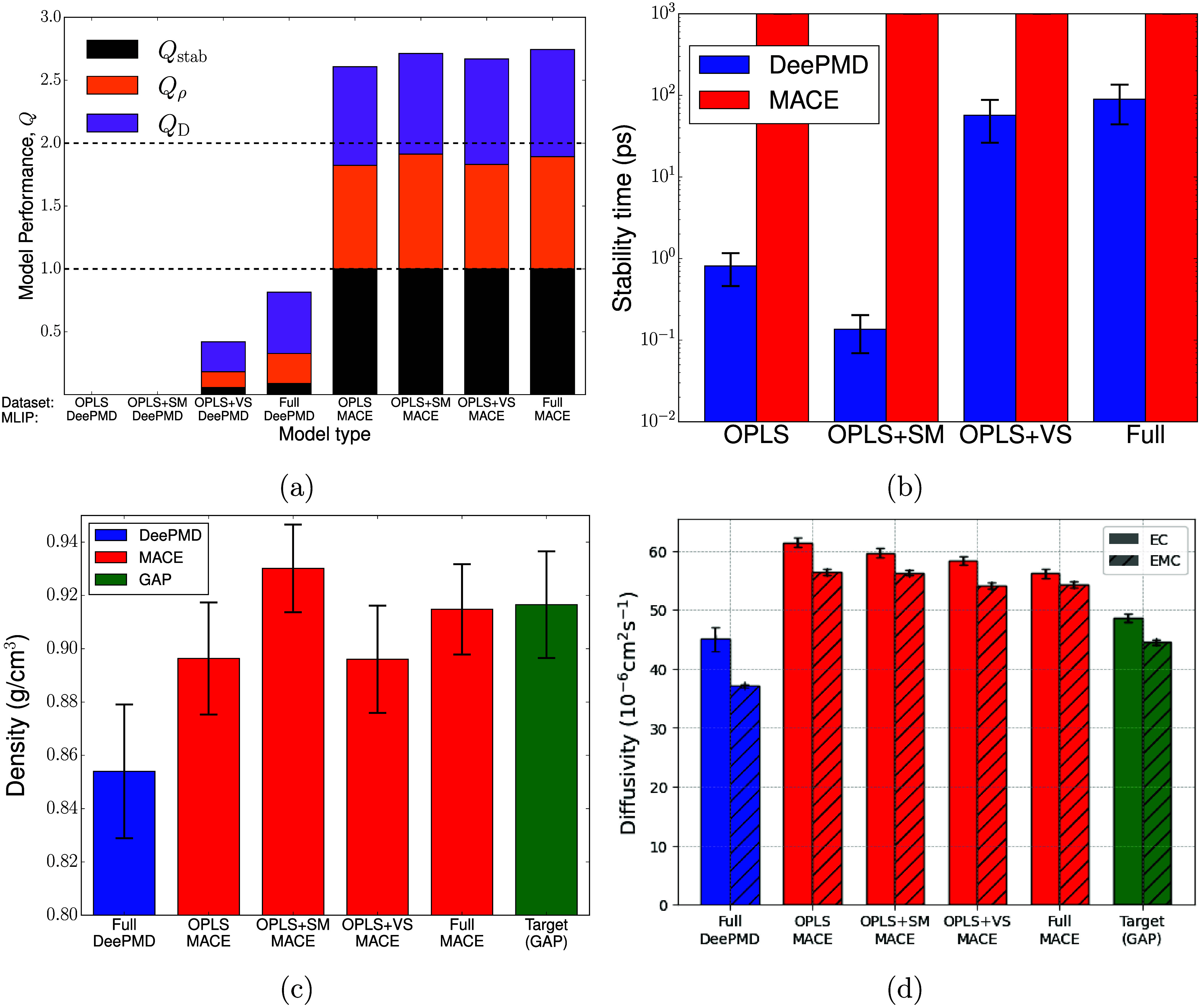
Measures of performance for models trained on transferred data alone. All MD simulations were performed at 500 K for 640-atom systems (1:2 EC/EMC by mole). In panels (b–d), bar colors indicate MLIP architecture which is also given on the abscissa for most panels. (a) Quality metric defined in eq 1, evaluated for each training set and MLIP architecture. (b) NPT stability time, *t*_stab_, for DeePMD and MACE models. Error bars represent a standard error across 5 equivalent models. (c) Predicted mean densities for selected MLIP committees, as labeled on the abscissa. Only models whose trajectories were stable for >100 ps are included in the average, and the first 100 ps of each trajectory were discarded for equilibration. The green bar labeled Target represents the density of the reference GAP model.^17^ Error bars indicate the standard error across the set of stable models. (d) Predicted canonical-ensemble diffusivities for selected MLIP committees. Diffusivity is computed from a linear fit to the center-of-mass mean square displacement (MSD), averaged over a committee of up to 5 models (discarding those with stability <100 ps). Within each trajectory, the MSD was averaged over a set of overlapping 100 ps blocks with starting points separated by 10 ps. An equilibration region of 100 ps was removed at the beginning of each trajectory and a ballistic regime of 10 ps was removed from each MSD block prior to the diffusivity fits. Error bars show the standard error of the average over all different blocks. Of the DeePMD models, only the Full data set met the stability criterion used here.

In summary, *Q* < 1 indicates a model that does not provide stable liquid-state dynamics at atmospheric pressure, while values between 1 and 3 indicate stable models with increasingly good descriptions of the density distribution and diffusion coefficients of the liquid. We suggest that this metric provides a fairly complete and general approach to comparing diverse MLIPs (or indeed traditional force fields) and could easily be extended to other chemical systems.

Note that *Q* depends on system temperature and composition, but for computational convenience we have considered one state point (1:2 EC/EMC at 500 K). Developing MLIPs to capture a wider thermodynamic phase space would require different and larger training sets, but the fundamental connection between molecular interactions and collective properties remains the same across temperatures, so we anticipate that our conclusions regarding transferability between MLIP types will carry over to other conditions.

## 3 Results

### 3.1 Performance of Transferred Data Sets without Active Learning

To understand the effect of our different training data types, we first analyze the performance of DeePMD and MACE MLIPs trained using only components of the GAP data set (i.e., without iterative training using the MLIP algorithm under consideration). We compare properties for a single composition (1:2 EC/EMC, as discussed above) with a fixed cell size of 640 atoms (48 molecules). Note that, in line with common practice, this test cell is larger than the systems used for training. The test cell is still small enough that there may be significant finite-size effects, but these should be roughly constant since the cell size was equal for all validation trajectories.

Figure 2 shows model performance using the metric and key properties identified in Section 2.2. Panel (a) shows greatly improved performance for MACE models compared with DeePMD, in particular dramatically improved stability and density predictions. All four MACE models achieve *Q* ≈ 2.5, showing that they give reasonable descriptions of EC/EMC mixtures even for small training data sets. This data-efficient performance has been noted previously for message-passing MLIPs.^41^ *Q* values do not significantly increase on expanding the data set with further transferred data, suggesting that the OPLS configurations already contain most of the necessary structural information for a good MACE model. By contrast, DeePMD models are usually unstable when trained on transferred data alone, with only a few trajectories surviving long enough to compute the density and diffusivity information. The largest training sets, particularly those which include volume-scan data, clearly perform much better than the small OPLS-based data sets. This important conclusion is discussed further below. Some DeePMD models with the largest data sets give reasonably accurate density and diffusivity predictions, but the committee-averaged *Q*_ρ_ and *Q*_D_ remain lower than MACE because other members of the committee are highly unstable. Further iterative training is therefore necessary to obtain reasonable performance with the DeePMD model.

Figure 2b shows the stability behavior of the models more clearly. MACE models rarely suffer trajectory failure for any reason, which we attribute to the larger receptive field, higher body-order, and greater smoothness of the MACE potential, all of which reduce the probability of encountering undertrained configurations during a typical MD simulation. DeePMD models trained on OPLS configurations alone do not yield a stable liquid: most trajectories fail after less than 100 ps, typically in a catastrophic event where molecules break apart and energy conservation is lost. This instability is likely a consequence of model overfitting due to small training set sizes: the DeePMD models give small force errors in well-trained regions of configuration space but have many “holes” where the potential is underdetermined and the errors are high. Note that ref 17 reports that GAP models trained on OPLS data alone were also unstable in NPT simulations. MACE, which has a much lower threshold for the required training data, does not suffer from this problem.

Most DeePMD trajectory failures involve breakdown of intramolecular geometry, but expanding the training data with single-molecule configurations that explicitly sample intramolecular coordinates (i.e., the OPLS + SM data set) actually decreases stability. In contrast, incorporating the volume scan configurations increases *t*_stab_ by 2 orders of magnitude. These results suggest that our DeePMD trajectories typically fail when two molecules approach unphysically close distances, leaving the well-trained region of configuration space and thus facilitating unrealistic breakdown of the molecular geometry. Volume scan configurations, that sample local densities up to 2 g/cm^3^, can teach the model to avoid this close-approach and thus reduce the failure rate. This insight is valuable for training future MLIPs in the low-data regime, since volume scans are a cheap and efficient way to augment a training set (with few additional reference DFT calculations required), thus offering a rapid route to stable initial models and reducing the requirement for iterative training.

All MACE models predict a mean density within the error of the target value (Figure 2c). Again, the many-body descriptors and smoothness of the MACE potential permit accurate property prediction even from small training sets that are statistically different from the DFT structural ensemble. The best-performing DeePMD models achieve slightly worse performance.

Figure 2d presents canonical diffusion coefficients calculated for the center-of-mass coordinates of EC and EMC molecules. We show only those models that were sufficiently stable to compute diffusivities. The Full DeePMD model gives a slight underprediction of the diffusivity relative to the reference value, while the MACE models exceed the reference by 10–20%. This deviation is consistent across all four data sets for both EC and EMC, and significantly exceeds the statistical error. It appears to be a systematic effect of the MACE potential form, but it is unclear whether the MACE diffusivity is more or less accurate than the DeePMD and Target values. As mentioned previously, the Target value was benchmarked against experimental and DFT density data but not against diffusivities, which severely complicates assessing model accuracy in this area.^17^

Given that MACE models have lower validation errors and a larger receptive field than GAP (12Å vs 6Å), we hypothesize that the MACE values presented in Figure 2 are actually closer to the DFT diffusivities than the GAP reference MLIP. We attempt to confirm this expectation in Section S4 by evaluating model errors over the course of NVT trajectories for MACE and DeePMD MLIPs. This approach aims to quantify how accurately the models describe the configuration space that they explore and that determines their predicted properties, thus assessing the reliability of those predictions. The DeePMD model explores increasingly high-error configurations over the course of the simulation (and these errors significantly exceed the validation error during training), whereas MACE remains in its well-trained configuration space. This test is not definitive but is more thorough than typical methods of assessing MLIP performance. It leads to the surprising conclusion that MACE models trained on a small classical data set provide more reliable dynamic properties than GAP models with a carefully crafted iterative training data set. This difference is probably because the intermolecular energy barrier configurations, on which diffusivity depends, are difficult to capture in a training data set without careful targeted sampling. Therefore, a model with good extrapolative ability and a simple data set, like MACE, is able to outperform poorly extrapolating models trained on complex equilibrium data sets. These results demonstrate why MACE is suitable for developing foundational models.

Figure 2 allows us to consider one of our key questions: whether configurations that were obtained by iterative training using the GAP MLIP can transfer successfully to other models or whether they are specific to the dynamics and holes of their parent model. The main difference between the Full and OPLS + VS data sets is the inclusion of these iterative configurations, so comparing these models should indicate the extent of their transferability. Note however that the additional iterative configurations also span a range of EC/EMC compositions, so we cannot completely separate the effect of iteratively generated configurations from the effect of incorporating multiple compositions into the training set.

DeePMD models trained on the Full data set are only slightly more stable than those trained on OPLS + VS: the mean stability time increases by around 40%, comparable to the statistical error in *t*_stab_. Both training sets yield a predicted density of 1.02 g/cm^3^, slightly higher than the reference value. A difference between these models emerges when we consider the diffusivities: the species-averaged diffusivity predicted by the Full models (39 × 10^–6^ cm^2^/s) is significantly closer to the reference value (48 × 10^–6^ cm^2^/s) than that predicted by OPLS + VS (23 × 10^–6^ cm^2^/s). However, this difference may simply be because the OPLS + VS models are only marginally stable (*t*_stab_ ≈ 100 ps), so extracting an accurate diffusivity is challenging.

The fact that stability hardly improves on going from OPLS + VS to the Full data set indicates that the high-error configurations leading to failure in the DeePMD models are qualitatively different (or at least distant in feature space) from those in the GAP model. This idea will be tested further in subsequent sections. Our results suggest that model-specific configurations produced by iteratively training a GAP MLIP provide little benefit to DeePMD models, except possibly by reducing random force errors and hence improving the predicted diffusivity. Given the similar expressibility of the GAP representation compared with DeePMD, we anticipate that transferring DeePMD-iterative configurations to GAP would be similarly unhelpful.

An equivalent conclusion is hard to draw for the MACE models since the stability is high for all training sets. This robustness is a key advantage of the MACE architecture, suggesting that classical force field data alone are sufficient to circumvent many iterations of active learning when using MACE. Although all MACE predicted densities are within the error bar of the reference value, those from the Full models are remarkably close to the reference value compared with other data sets. This closeness may indicate that MACE models benefit from including GAP iterative-training configurations and multiple molecular compositions. The higher body-order and larger receptive field of the MACE descriptors may allow them to learn from such configurations even if these are not close to the “holes” that need to be corrected in the MACE configuration space, thus allowing greater transferability of data than in DeePMD MLIPs.

### 3.2 Effect of Transferred Data on Active Learning Performance

Notwithstanding the considerable success of the MACE models even for very small training sets, it is instructive to consider how much active learning effort may be saved by reusing existing training configurations. We therefore assess how fast DeePMD models with different initial training sets improve during an active learning procedure. We develop an active learning protocol intended to represent common practice in the field, details of which are provided in Section S3.

Figure 3 shows how each measure of performance changes with number of active learning iterations, for four families of models labeled by their initial training sets. Thus, the iteration-0 data in this figure correspond to the models shown in Figure 2. The key features of this figure agree with the conclusions of the previous section: reusing volume scan configurations provides a major increase in stability and hence overall performance, but there is little or no benefit to reusing iterative-training data.

**Figure 3:**
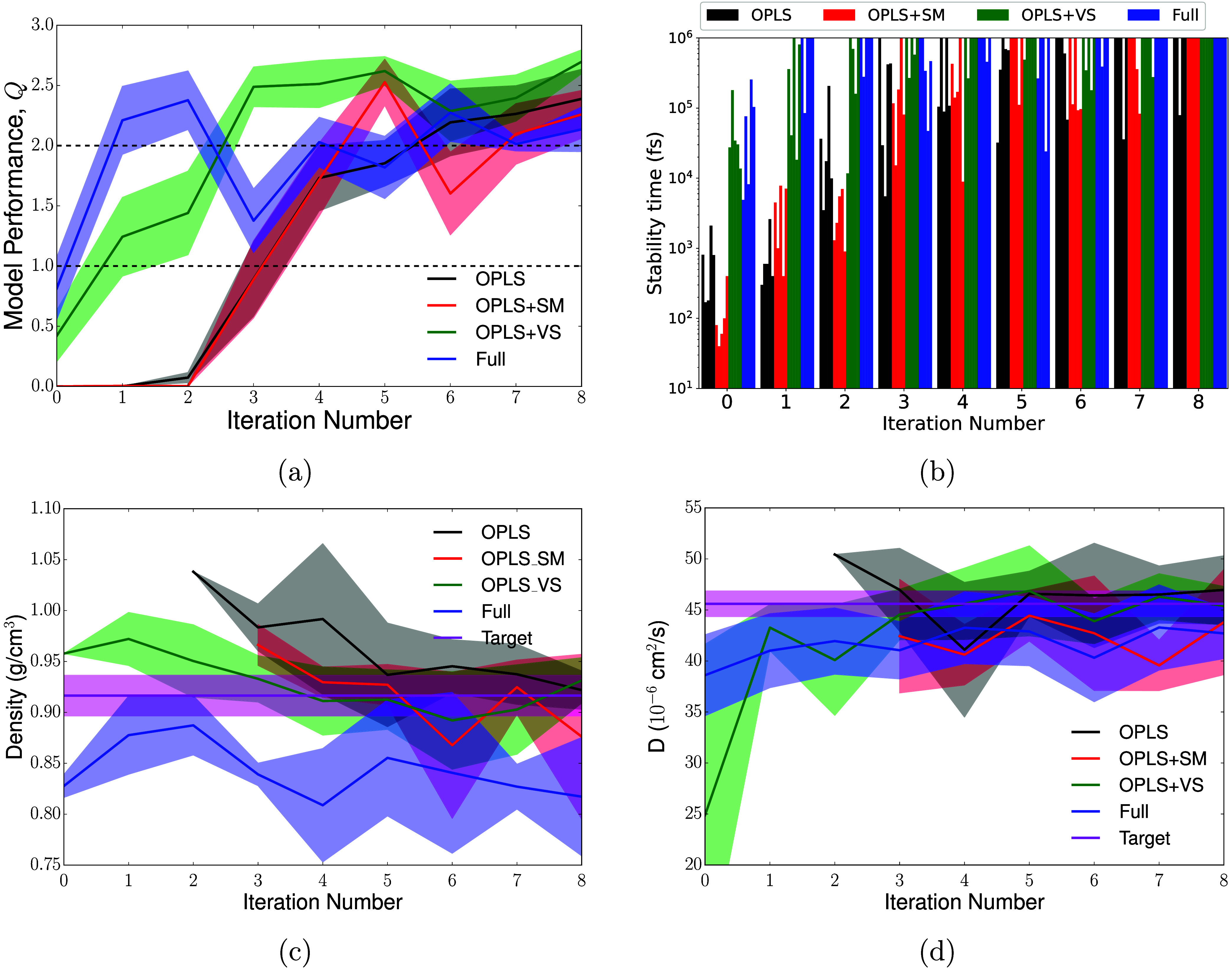
Performance of DeePMD committees that start from different data sets over a series of active learning iterations. In panels (a), (c) and (d), solid lines represent committee-averaged quantities, and shaded regions represent the standard error of this mean. (a) Overall model quality, as defined in eq 1, as a function of active learning iteration number. (b) NPT Stability times (defined in Section 2.2) as a function of active learning iteration. Each bar represents a different MLIP model, all models with the same color use the same initial training set with different random seeds. (c) Predicted density as a function of active learning iterations. Only models with *t*_stab, NPT_ > 0.1 ns are included in the committee analysis, and the density is averaged across the interval [0.1 ns,*t*_stab, NPT_]. (d) Molecule-averaged diffusivity as a function of active learning iteration. Diffusion coefficients were computed as described in the caption to Figure 2(d), but for clarity, we show only the average of the EC and EMC diffusivities (weighted by their mole fraction). Note that the error in this case is the standard error across a committee of model predictions rather than across trajectory blocks as in Figure 2(d).

Panel (a) shows that the performance metric *Q* (eq 1) increases with successive active learning iterations for all models, particularly a rapid increase that occurs after *t*_stab_ reaches 1 and all members of the committee are able to generate stable density predictions. This increase occurs between iterations 1 and 3, depending on the initial data set. It appears that the first iterations of active learning largely teach the models to avoid pathological failures, and only once these “holes” are corrected do new training configurations begin to improve the density and diffusivity predictions. Panel (a) also shows that initiating active learning with more transferred data generally decreases the number of iterations required to reach higher performance. Incorporating volume scan configurations yields the greatest improvement in *Q*, while single-molecule configurations offer little or no benefit relative to the OPLS-only models.

A key result of this panel is that reusing the Full GAP data set provides no long-term benefit compared with OPLS + VS. A small initial improvement results from the mildly increased stability and more accurate diffusivity predictions. However, the density predictions of the Full model actually worsen with successive generations of active learning, so that by generation 8 this committee has a *Q* no higher than the other training data sets. Possible reasons for this behavior are discussed below.

All four models achieve a similar performance after about 6 iterations of active learning, which suggests that the liquid properties of a converged model are mostly controlled by the algorithm-specific active-learned configurations rather than the data transferred from GAP. However, the latter are still helpful for reducing the amount of training required to reach this performance.

Panel (b) shows that the rate of stability improvement during active learning mirrors the trend in stabilities at generation 0: the models with the most transferred data (and hence the highest initial stability) quickly improve, reaching *t*_stab_ ≈ 1 ns after 2–3 generations. The data-poor models (OPLS and OPLS+SM) take significantly longer to achieve this stability, typically 6–8 generations. This difference indicates that OPLS and OPLS + SM have more “holes” in their well-trained configuration space that must be fixed by active learning in order to improve stability. It is also possible that these models’ unstable initial trajectories generate configurations which are so dissimilar to the target configuration space that training on them does not improve model accuracy for the near-equilibrium configurations where trajectory failures occur. By contrast, models initialized with larger data sets (OPLS + VS and Full) remain closer to the target configuration space, so training on the high-disagreement configurations selected from these trajectories helps prevent the close-molecular-approach events that initiate trajectory failure.

Each DeePMD model initially predicts a mean density substantially different from the target (see panel (c)). For most data sets, this difference shrinks over successive generations, usually accompanied by a reduction in the standard error as the different members of the committee begin to converge on the same density value. These results confirm the utility of transferring volume-scan data, which both improves stability and reduces the number of active-learned configurations required to converge the liquid’s properties. The Full model here is an outlier, with predicted densities remaining significantly below the target value, which is why its overall *Q* falls below that of other models, as previously noted. This deficiency will be discussed further in the next section.

Turning to panel (d), we see that DeePMD model diffusivities agree quite closely with the GAP values: most stable models predict a diffusivity within one standard committee error of the target, regardless of their training data or the amount of active learning. However, the committee error is quite large and does not significantly decrease during active learning. This close agreement suggests (counterintuitively) that canonical diffusivity is less sensitive than density to errors in configuration energies. Diffusion rates depend strongly on high-energy ”barrier” configurations that control which regions of configuration space are visited by the trajectory and how fast these regions are explored. Barrier configurations are usually underrepresented in MLIP training data and so have large prediction errors; thus, we expect that diffusivity should be initially poor and converge slowly to the correct value during active learning. This behavior is not observed, with model predictions starting and remaining close to the target value and converging equally quickly as the predicted densities. This observation may indicate that the diffusivity in this system is largely determined by the liquid density, with only a minor contribution from the energy barrier heights. Recall that our diffusivities were computed using NVT simulations at a fixed density equal to the GAP reference value, precisely in order to control for the effect of the erroneous model density on the diffusion coefficient. For flexible organic liquids, such as EMC, it is reasonable to expect that density will be closely correlated with viscosity and hence strongly influence diffusion. Possibly, this effect is dominating over that of varying energy barrier heights and producing similar diffusivities for all models. We expect that further active learning will reduce the barrier errors and hence the committee disagreement in the diffusivity. But evidently, we require significantly more iterations to achieve this reduction, and testing this effect is beyond the scope of the current contribution.

Note also that the uncertain accuracy of the reference diffusivities compared with MACE predictions (see previous section) means that we are unable to draw strong conclusions from the generally good agreement between DeePMD and GAP in this panel.

### 3.3 Understanding the Information Content of Transferred Data Sets

The previous section showed that different components of the GAP data set provide varying benefits when transferred to MACE or DeePMD. In particular, volume-scan configurations dramatically increased the stability of DeePMD models. We now probe the specific limitations and utility of each data type further, focusing on naive models trained only on transferred data (i.e., without active learning). Specifically, we consider how well the MLIPs trained on each transferred data set are able to describe energy variations with changing density. The previous discussion emphasized the difficulty of predicting density correctly and hypothesized that avoiding large underpredictions of the energy at high density is essential to avoid catastrophic trajectory failure (at least in DeePMD). Therefore, this energy variation is an appropriate additional measure of model quality. It also provides some insight into why each class of models performs as described in the previous section.

Figure 4 shows how different DeePMD and MACE models predict that the energy of a small EC/EMC cell varies under isotropic compression or expansion at constant intramolecular geometry—i.e., a volume scan, the same as those used to generate the VS configurations in the GAP training set. A volume scan provides a validation set that is constant for all models and where the relationship between the different configurations is clear and known. Any configuration of the liquid can be used to generate a volume scan, producing different energy curves in each case.

**Figure 4:**
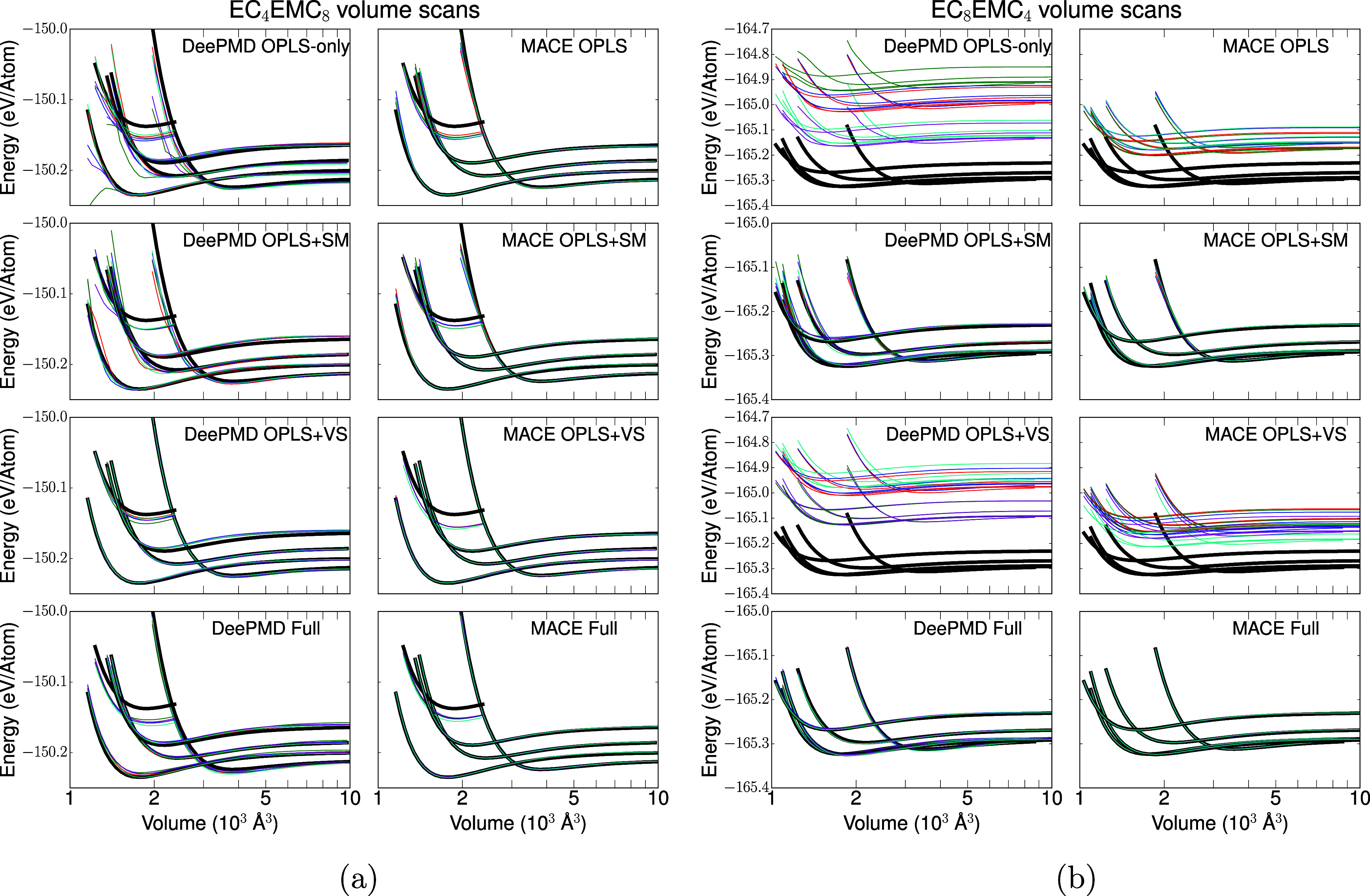
Potential energy predictions for DeePMD and MACE models trained on different transferred data sets without active learning, evaluated for volume-scan configurations. Each curve corresponds to the energy of a particular molecular arrangement during isotropic expansion and compression. In each panel, the left and right columns show DeePMD and MACE models, respectively. (a) Scans for simulation cells containing 4 EC molecules and 8 EMC molecules and (b) EC_8_EMC_4_ cells. Each subpanel is labeled with its training set according to the convention in Section 2.1, and each contains volume scans of 5 different configurations at the given composition. Thick black lines indicate the DFT energies for each scan, and each colored line represents predictions from one model out of a 5-member MLIP committee.

In all panels of the figure, the energies diverge at small volumes due to close-approach of atoms and tend to a finite plateau in the large-volume limit where every molecule becomes noninteracting. Validating an MLIP during a volume scan tests both how it describes environments at nonequilibrium intermolecular distances and also the intramolecular energy of an isolated molecule.

We compare volume scans for 5 configurations of a 1:2 EC/EMC liquid and 5 configurations of 2:1 EC/EMC. Two types of prediction failure are immediately apparent. In several subpanels, the shape of the MLIP energy curve deviates significantly (around 0.05 eV/atom) from the DFT value at low volumes. These deviations probably correlate with the “holes” noted in previous sections that are responsible for catastrophic trajectory failure. In particular, note that many of the DeePMD OPLS and OPLS + SM curves have large energy errors at low volumes, permitting the unphysically close-approach of neighboring molecules and making the corresponding trajectories very unstable. Unsurprisingly, these deviations decrease for the data sets that include volume scan training data, in line with their improved model performance noted previously. They are also not observed for MACE models, which have smaller errors in general and which show well-behaved close-approach curves even for the smallest data sets.

The second prediction error affects both DeePMD and MACE models and takes the form of a large energy shift (up to 70 eV, 0.5 eV/atom) relative to the DFT value. This shift is roughly consistent for all scans with a given model and roughly constant across the volume scan. This error occurs in the 2:1 composition curves and arises because models trained on a single composition (1:2 EC/EMC) cannot learn correctly how the baseline energy of the noninteracting limit should be partitioned between EC and EMC molecules. Therefore, when the composition changes, the energy baseline shifts by an incorrect amount. This baseline issue is potentially serious for large simulation sizes, where individual atomic environments may sample very different local compositions over time, resulting in large fluctuations in the total predicted energy that could prompt unphysically large responses from thermostats (for example).

The issue of incorrect energy partitioning has been discussed by Goodwin et al.,^41^ who showed that it can be corrected by training on multiple compositions of the system. The final row of panels (full data set) demonstrates this improvement very clearly. The second row of panels demonstrates that the erroneous shift is also easily corrected by training on configurations containing isolated molecules (OPLS + SM). Training on volume scan data (OPLS + VS) does not contain information about the correct partitioning of molecular energies, so the incorrect baselines persist in those panels of Figure 4.

Incorporating isolated-molecule configurations into a training set is therefore an efficient strategy to improve transferability between compositions. However, recall that Section 3.2 shows that this approach may not improve trajectory stability. In our system, the baseline energy shift may also be removed by adding a single isolated-EC configuration and one isolated-EMC, as long as these configurations were assigned high selection weights so that each was included in 5% of training batches. This is a simple and computationally efficient way to augment a training set to capture energy partitioning. However, the resulting model has higher prediction errors than models that use many isolated-molecule configurations (i.e., the complete OPLS + SM set). Therefore, including further training configurations, such as volume scans or transferred iterative configurations, is also necessary to achieve stable liquid dynamics.

The two DeePMD Full subpanels show an interesting trend: in contrast to every other DeePMD data set (and to all MACE models), the prediction errors are slightly worse for 1:2 EC/EMC than for the 2:1 liquid. In Figure 5(a),(b), we further show that errors in 1:2 EC/EMC for the Full model are larger than those for the OPLS + VS model, particularly for the highest-energy volume scan. We hypothesize that this difference arises because the additional training configurations present in the Full set span a range of compositions, thus improving the MLIP’s description of 2:1 EC/EMC while decreasing the training weights of 1:2 liquid configurations. In this view, the larger compositional phase space that the Full models attempt to learn results in higher errors for the specific composition region that we then test. This deficiency may account for the persistent underprediction of densities in the Full model during active learning (Figure 3c), which was also tested only on the 1:2 EC/EMC composition.

**Figure 5:**
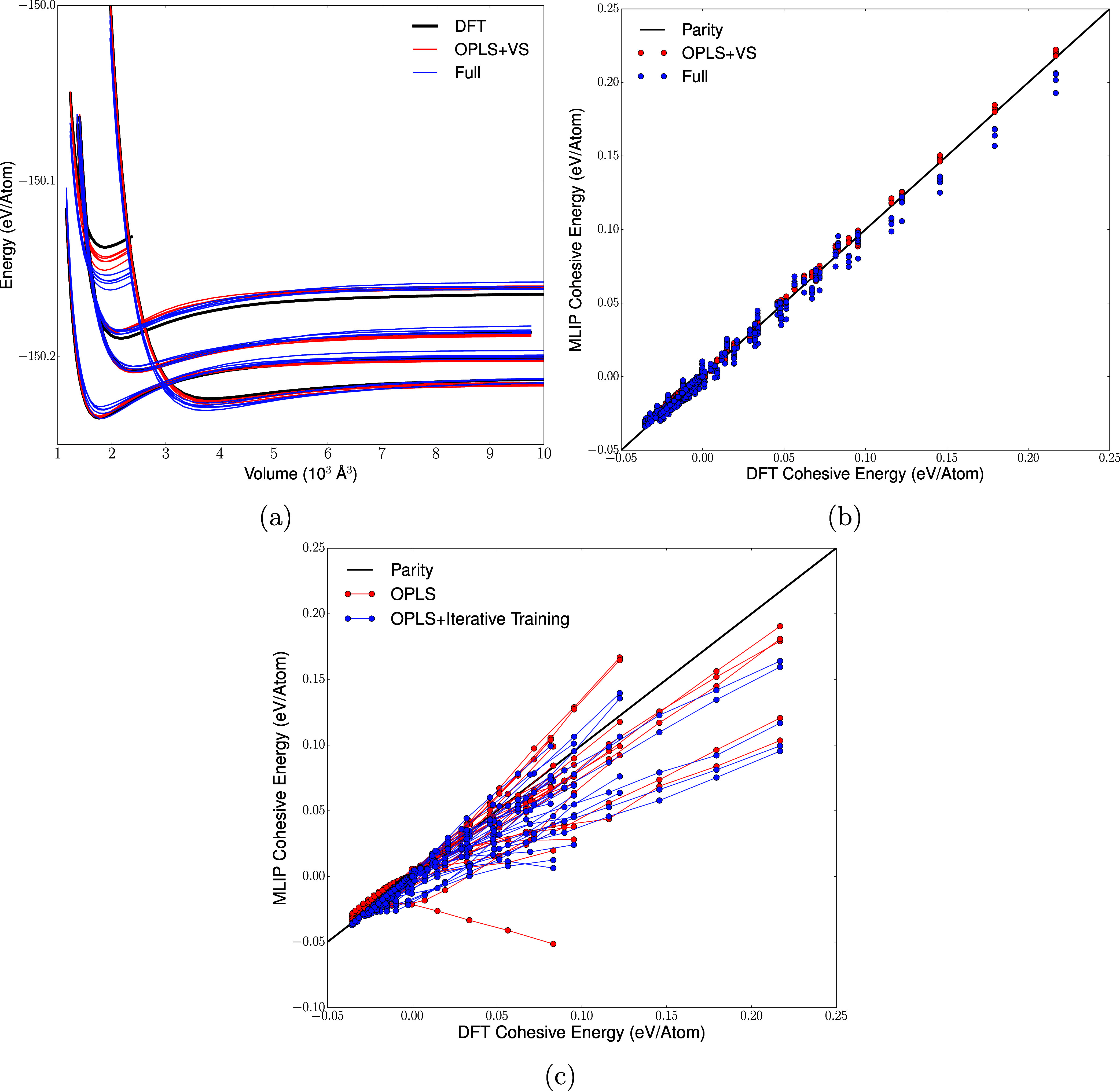
Further details of volume-scan tests on DeePMD models. All panels refer to tests with a 1:2 EC:EMC composition. (a) Energy as a function of cell volume for two committees of models, trained on the OPLS + VS and Full data sets, compared with DFT. (b) Energy prediction parity plot for volume scans of models trained on OPLS + VS and Full data sets. The black line indicates perfect prediction. This plot aggregates predictions from all 5 members of the model committees across all 5 volume scan configurations. Cohesive energies are shown rather than absolute energies to allow comparing different volume scans (that have different intramolecular energies). The cohesive energy is calculated by subtracting the large-volume energy limit (evaluated using either DFT or the MLIP, as appropriate) from the whole volume scan. (c) Parity plot comparing models trained only on OPLS configurations with models trained on OPLS plus the subset of GAP iterative-training configurations that a composition of 1:2 EC/EMC. Adjacent points on a volume scan are joined by lines to show the relationships between them.

This observation suggests that correctly describing a range of compositions using a simple neural-network-based MLIP requires much larger data sets and likely many more training epochs than if one is interested only in a single liquid composition. Increasing the flexibility of the MLIP architecture (e.g., the size of the neural network) might ameliorate this problem, but would require much more training data to work effectively. As a corollary to this argument, we suggest that the slightly higher stability of the Full models relative to OPLS + VS (observed both with and without active learning) arises more from their ability to describe a variety of local liquid compositions than from the inclusion of GAP-iterated configurations in this data set. More numerical work would be required to test this suggestion, however.

Figure 5c shows a parity plot based on further volume scan tests which reinforces our earlier conclusion that iterative configurations transfer poorly from GAP to DeePMD. This panel uses a new transferred data set combining the OPLS data with the subset of GAP iterative-training configurations that have the 1:2 EC/EMC composition, thus isolating the effect of iterative training alone. No substantial error reduction, in either magnitude or bias, results from including these configurations compared with the OPLS-only data set. This result does not necessarily indicate that the GAP active-learned configurations offer no improvement to the model at all since they may help to prevent MD trajectories from visiting unphysical regions of configuration space, but they confer no specific advantage in describing high- or low-density configurations as measured by the volume scans.

## 4 Further Aspects of Data Reuse

In this section, we present two preliminary studies into the possibilities offered by reusing training data. The first compares our bespoke transferred models with pretrained foundational MLIPs, which represent the fastest route to obtain MLIPs for a novel system. The second study concerns the ability of a transferred-data model to generalize into unseen chemical systems. These sections do not represent complete investigations but are intended to illustrate the scope and limitations of data reuse as a training strategy, as well as motivate further study in the respective areas.

### 4.1 Performance of Pretrained Models

To understand the importance of system-specific training data and to explore the potential value of data reuse in conjunction with FMLIPs, we tested the performance of two pretrained MACE models for our EC/EMC test system: MACE-MP-0 ^27^ and MACE-OFF.^48^ The former is an extremely general foundational MLIP that aims to describe atomic and molecular materials across the periodic table. It is trained on the Materials Project database, which uses PBE DFT energy calculations as a reference (similar to our own models). MACE-OFF is designed to simulate organic and biological molecules, and is trained on a subset of the SPICE data set with energies calculated at the ωB97M-D3­(BJ)/def2-TZVPPD level of theory (which is significantly more accurate but also more expensive than PBE).

MACE-MP-0 is trained on PBE DFT energies with no dispersion forces, and we find that liquid trajectories with this model evaporate rapidly. Adding a Grimme D3 dispersion correction^52^ to the MLIP energies and forces yields a stable liquid with a predicted density of approximately 0.72 g/cm^3^. This prediction is significantly worse than the best-performing DeePMD and MACE models trained on bespoke EC/EMC data, indicating that fine-tuning on system-specific configurations would be required to use this model practically for organic liquids. We note in passing that the underpredicted density and need for fine-tuning are both consistent with recent findings that foundation model energies and forces are often systematically “soft”, with a relatively small number of high-energy training configurations required to correct them.^31^ We therefore anticipate that MACE-MP-0 fine-tuned with a few OPLS configurations (similar to our MACE OPLS model) should perform very well for this system.

The higher level of theory used to train MACE-OFF means that it is not directly comparable to our reference data. This MLIP model predicts evaporation of the EC/EMC liquid at around 400 K, which is probably much closer to experimental reality than the PBE-D2 models used elsewhere in this work (pure EC boils at 516 K and EMC at 380 K). The mean density of the model at 300 K was 1.15 g/cm^3^, fairly close to the experimental value of 1.09 g/cm^3^,^17,53^ but we cannot currently determine whether the remaining variance results from stochastic training errors or from the DFT functional used to train MACE-OFF. Unsurprisingly, for a model specifically and carefully trained to describe organic liquids at a high level of theory, MACE-OFF predictions appear to be more robust than MACE-MP-0 and more faithful to experiment than the purpose-trained MACE and DeePMD models presented earlier. MACE-OFF would likely be a suitable surrogate to generate initial training data for future bespoke models (even if these configurations are recalculated at a lower level of theory for computational convenience). However, it is hard to assess how well this model would perform when tested outside of its training data (e.g., for a practical electrolyte containing ions).

### 4.2 Effect of Transferred Data on Chemical Generalizability

Many applications of MLIPs, particularly in materials chemistry and chemical biology, benefit from a degree of generalization to different molecules to avoid the expense of training multiple models when studying a series of related chemicals. This generalization is particularly important when screening candidate materials, in our case candidate electrolyte formulations, for desired properties.^34^ Here, we investigate whether different amounts of transferred training data have a significant impact on the generalizability of the resulting model.

We analyze the ability of our EC/EMC DeePMD and MACE models to model three different chemical systems: EC/DEC 1:1 v/v, VC/EMC 1:1 v/v, and pure PC. DEC is diethyl carbonate, VC is vinylene carbonate, and PC is propylene carbonate; their chemical structures are provided in Figure 1. All three molecules are used as battery electrolyte components, and each tests a different aspect of MLIP generalization. EC/DEC is a popular solvent for sodium-ion batteries,^54^ and chemically almost identical to EC/EMC. PC is used in various batteries as either a single solvent or cosolvent; it has greater thermal and oxidative stability than EC, which means that it does not form a thick passivating layer on graphitic anodes^55^ and is therefore prone to cointercalation with Li inside those anodes. PC is chemically similar to EC but contains an additional methyl group not found in the original EC/EMC training data, so this molecule tests the ability of an MLIP to generalize molecular shapes and also to describe a unary liquid as opposed to the binary mixture on which the MLIPs were trained. VC is structurally similar to EC but chemically distinct, since it possesses a carbon–carbon double bond. It is used as an additive to a variety of battery solvents, and is believed to form more stable solid-electrolyte interphase layers,^56^ its double bond promoting formation of branched polymeric structures. Our test liquid has a much higher VC concentration than is typically used practically, to ensure that any generalization errors of the MLIP are prominent and easy to measure.

For these test molecules, we had no high-quality AIMD data against which to compare, and hence no reference values for the density or diffusivity. Instead, our analysis focuses on stability time as previously defined and on analyzing the prediction errors against DFT for a sample of configurations drawn from the MLIP trajectories.

For each molecular system, we tested the four committees of MACE models that were analyzed in Section 3.1, four committees of DeePMD models trained on the same data, and four committees of DeePMD models that had received active learning on the EC/EMC system. These DeePMD models correspond to generations 0 and 8 of the active learning scheme in Section 3.2.

Figure 6a shows the NPT stability times for each of these models. A strong relationship is seen between EC/EMC stability and EC/DEC stability: MACE models are highly stable, DeePMD models without AL are unstable but improve with increasing training set size and particularly with volume-scan configurations. This result is unsurprising as DEC contains no structural motifs that are not present in EMC, hence the atomic environments present in this liquid probably have similar descriptor functions to those of the models’ training data. Likewise, it is not too surprising that EC/EMC active learning improves model stability for EC/DEC. Note however that the DeePMD + AL Full model is slightly less stable and has a higher error than the other models, which may be another consequence of the more diverse initial training set, as discussed previously.

**Figure 6:**
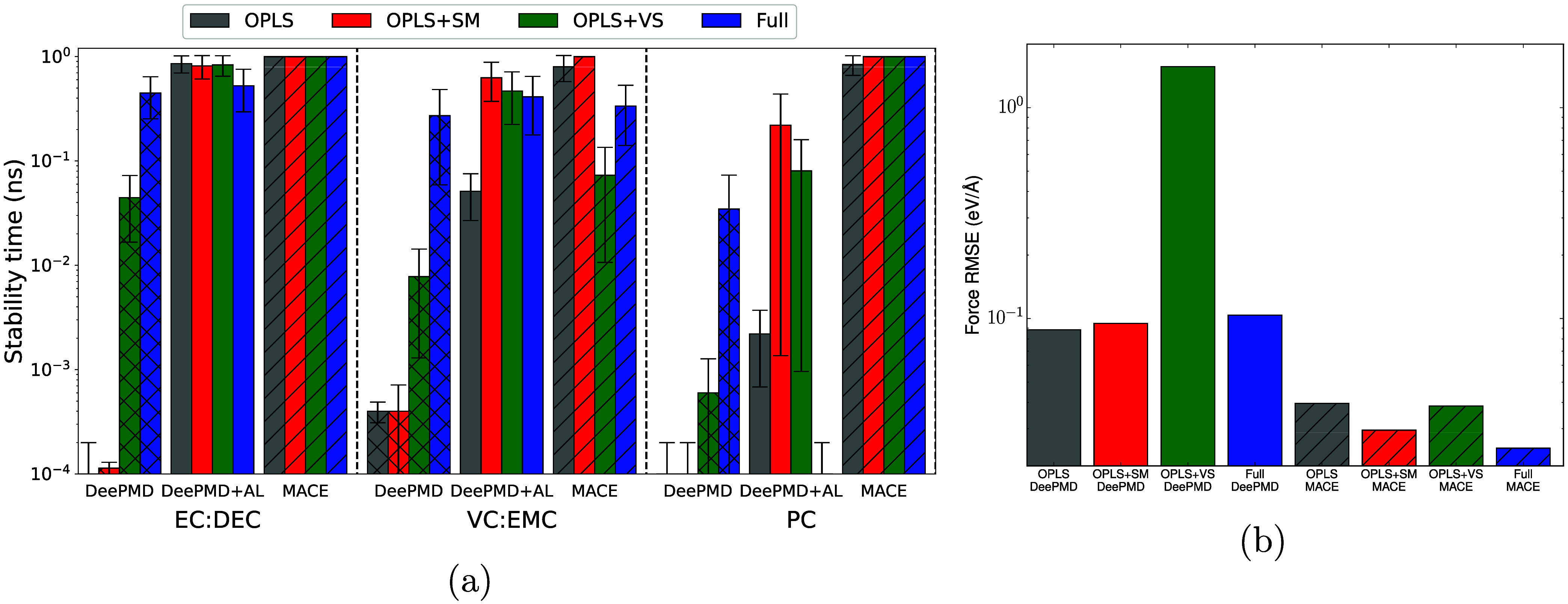
Comparison of different model performances when generalizing to untrained chemical systems. In both panels, colors correspond to different initial training sets and hatching indicates model type. (a) NPT stability times for DeePMD and MACE models evaluated on different chemical systems. Test systems and model types are indicated on the horizontal axis; “DeePMD” denotes DeePMD models trained only on the indicated data set; “DeePMD + AL” indicates that the data set also includes 8 generations of active learning on EC/EMC configurations as discussed in Section 3.2. MACE models were trained on the initial data set alone. (b) RMS errors (vs DFT) in force predictions for EC/DEC liquid for MACE and DeePMD + AL MLIPs. These errors were computed over a sample of configurations from the trajectory of the model in question rather than from a fixed test set. Plain bars indicate DeePMD + AL models; hatched bars represent MACE.

Model performance in EC/DEC is further examined in Figure 6b, which shows force prediction errors versus DFT for configurations sampled from a stable NPT trajectory. With the exception of an anomalously large error for DeePMD + AL with the OPLS + VS training set, which may indicate that this particular trajectory entered an extrapolative region of configuration space without failing catastrophically, all bars are comparable with the corresponding EC/EMC force errors (approximately 0.1 eV/Åfor DeePMD and 0.025 eV/Åfor MACE)—again suggesting good generalization from EMC to DEC.

VC molecules contain a functional group not present in the training sets (the alkene moiety), which our MLIPs are mostly unable to describe correctly. Panel (a) shows that most MLIP trajectories did not meet the 1 ns stability criterion, typically due to unphysical breaking of a C–H bond or the C=C double bond in an attempt to create an EC-like geometry. We expect that all models would ultimately fail in this way, but for some, the kinetic barriers to bond-breaking are large enough that they remain stable for 1 ns. Variations in performance between DeePMD + AL and MACE, or between different training sets, were small and inconsistent for this molecular system. However, the clear improvement in DeePMD stability after EC/EMC active learning is striking and indicates that configurations that correct deficiencies for the EC/EMC liquid are also helpful to correct errors in this liquid. Presumably, the additional training prevents a large fraction of failure events involving atomic environments that are common to both molecular systems, leaving only cases where a trajectory fails due to poor prediction of the alkene-carbon energies and forces.

The PC liquid shows a clearer difference between the DeePMD and MACE models: the latter remain stable for long periods, while most of the former fail through unphysical reactions within 100 ps. These results indicate that the MACE descriptors recognize the chemical similarity of EC and PC, where the DeePMD descriptors do not, presumably because the former takes account of higher-order correlations that facilitate distinguishing between branched and linear structures. Once again, the stabilities of most DeePMD models are significantly improved by active learning, which indicates that similar pathological configurations cause trajectory failure in PC as in EC/DEC. We previously argued that these pathologies mostly arise from an unphysical close-approach of distinct molecules. Including volume scans and running targeted active learning to suppress these high-density local environments is therefore a useful strategy for improving the generalizability of an MLIP, as well as performance on its native chemical system.

The notable exception to this rule is the DeePMD Full model: unlike the OPLS + SM and OPLS + VS data sets, EC/EMC active learning on the Full model produces no significantly stable committee members so that the post-AL bar is not visible on the scale shown in panel (a). We speculate that this deficiency is due to AL causing the model to overfit on the binary mixtures that constitute the active-learned configurations: the Full + AL model becomes so specialized to describing these mixtures that a strong tendency develops for some PC molecules to react and form a structure resembling the linear EMC. Such spurious “ring-opening reactions” have been observed anecdotally in our simulations. However, from the limited data we have presented, it is hard to say whether this model deficiency is general or whether it connects to the density underpredictions noted previously for the same model.

With that caveat, we tentatively conclude from this section that models trained on small bespoke data sets generalize fairly well to molecules that are chemically similar but structurally distinct. This is particularly true of the many-body MACE models. Moreover, active learning on the parent system usually improves generalization, a surprising but valuable result.

## 5 Conclusions

This paper has investigated the ability of MLIPs to describe organic molecular liquids, specifically battery electrolyte solvents, by using very limited training sets and data transferred from a different MLIP architecture. We have argued for the importance of validating models using their native dynamics rather than a fixed validation set and for comparing collective properties of the system (density, diffusivity) rather than per-atom properties such as energies and forces. We have demonstrated that these collective properties can be very challenging to predict correctly and depend strongly on the training data, since they are sensitive to the model’s description of both the low-energy and barrier regions of the potential energy surface.

Our most important conclusion is that MACE models perform well by all the metrics considered, even for small training sets that contain only classical configurations of the liquid, which suggests that this architecture is much less susceptible to “holes” (high-error regions in the learned configuration space) compared with earlier MLIPs. Our models were robust against failures in either intramolecular or intermolecular liquid structure and gave densities that agree well with the reference GAP model. The diffusivities predicted by MACE models were quite different from the reference GAP model, but simple tests indicate that MACE may be closer to the correct DFT diffusivities. We ascribe this excellent generalization ability to the large receptive field and high body-order of the MACE descriptor functions, which allow them to extrapolate properties well from small training sets. Understanding what property of MACE controls the reduction in holes is an important challenge for future MLIP development, and the data reuse paradigm may provide a valuable framework for testing this.

Simpler models such as feedforward neural networks and the GAP models studied previously^17^ require larger training sets to achieve good descriptions of a liquid, typically including multiple iterations of active learning. Models trained only on configurations sampled from a classical trajectory inevitably undergo unphysical reactions over very short time intervals. We find that these failures result from prediction errors in the intermolecular forces, rather than the intramolecular forces, and show that they can be largely corrected by training on volume scan configurations that are very cheap to generate. By contrast, transferring iterative-training configurations optimized for a different MLIP type (in our case, GAP) provides only minor improvements to model stability and properties, suggesting that the high-error “hole” configurations are quite different for DeePMD than for GAP—a result that is not surprising but is probably important for understanding the MLIP design space. Larger training sets (particularly those that include volume scans) do reduce the number of active learning iterations required to perform well, but we also saw indications that more diverse training sets may hinder the model’s ability to capture any one liquid composition accurately. We hypothesize that this reduction in performance resulted from the important low-energy configurations having lower training weights in these data sets, while the iterative-training configurations that are added provide no significant benefit. Further work is required to understand how this effect depends on the MLIP architecture and training hyperparameters.

Our DeePMD results suggest that data reuse between different MLIP algorithms saves little computational effort. However, the greater generalization ability of MACE may allow it to reuse training data more effectively; the smooth, long-ranged descriptors mean that each training point helps to define a much larger volume of configuration space than in the simpler MLIPs. MACE generalizes so well that our models can predict liquid properties accurately using only small classically derived training sets. This robust performance partially explains the recent success of the MACE architecture in the development of Foundational models.

Finally, we consider how models trained to describe one molecular liquid perform in simulations of a different chemical system. This question is important for effective collaboration with experimental work, which often involves rapid testing or screening of multiple molecular systems and is a requirement for reliable Foundational Models. Unsurprisingly, we find that small structural changes do not significantly impact model performance, but changing the functional groups degrades model stability significantly (both for DeePMD and MACE). Interestingly, model stability for unseen molecules correlates quite strongly with training set size, even improving on the introduction of configurations that were obtained by iterative training on the original molecular system. This result suggests that some trajectory failure events are a property of the MLIP architecture rather than specific training data or chemical application.

The generality of our results is potentially limited by the high computational cost of computing collective properties for organic liquids, which forced us to consider only one state point and liquid composition in detail (although our models were tested extensively on multiple compositions as part of the active learning procedure). Ongoing work in our groups is demonstrating that the high performance of MACE models carries over to other electrolyte systems and thermodynamic conditions, though it is hard to prove that the trends in data transferability identified here will hold for every scenario. We have also tested only data transfer from GAP to DeePMD and MACE, and not the converse. Our interpretation of these results suggests that neither GAP nor DeePMD will benefit from including iterative-training configurations for any other models, while MACE may see some improvement from DeePMD-derived configurations. Testing these hypotheses would be a valuable future study.

Overall, we conclude that conventional neural network and Gaussian process MLIPs remain poorly transferable and that any such model should be viewed as unique to both its underlying algorithm and its training data with little possibility of reusing data between algorithms. The situation for many-body message-passing models is more promising, in part because their data requirements are low enough to provide good performance without substantial active learning. Our results suggest that fine-tuning a foundational MLIP with a small amount of system-specific data (e.g., from a classical force field or pre-existing MLIP) will yield substantial improvements in performance. Equivalently, training data generated with a foundational model might be usefully transferred to a cheaper MLIP architecture, providing initial training sets that compromise between the accuracy of an AIMD calculation and the efficiency of classical MD. This possibility deserves further exploration.

Using the principles discussed in this paper to accelerate the development of MLIPs for molecular liquids will hugely advance materials chemistry and battery science. From exploring the effect of low-concentration additives on electrolyte chemistry to probing the formation and dissolution of solid-electrolyte interphase components, the unsolved questions in batteries are characterized by multicomponent liquids and materials. Allowing efficient *ab initio* quality models to be developed for many different molecule types without vast computational or human effort will open up these areas for simulation study and enable new modes of computational-experimental collaboration.

## Supplementary Material



## Data Availability

Research data required to reproduce the results and figures of this paper are provided via the Apollo repository at Cambridge: 10.17863/CAM.11585. This repository contains training data sets, MLIP model files, input and analysis scripts, and the summarized output of MD trajectory analysis. The full MD trajectories that were analyzed in the paper are available from the authors upon reasonable request.

## Notes

Comprising additional details of the MLIPs used, training protocols, and relative performance, as well as details of tests performed to assess the accuracy of diffusion coefficients computed using MACE and DeePMD (PDF)
