# Supplementary material for: Transferability of Data Sets between Machine-Learned Interatomic Potential Algorithms
Source: J Chem Theory Comput. 2025 Jun 5;21(12):6096–112. doi: 10.1021/acs.jctc.5c00272 (PMC12199462; doi:10.1021/acs.jctc.5c00272)
Supplement: Supplementary file 1 [file ct5c00272_si_001.pdf]

Supporting Information for: Transferability of datasets between machine-learned interaction potential algorithms

Samuel P. Niblett,[†] Panagiotis Kourtis,[‡] Ioan-Bogdan Magdău,[‡] Clare P. Grey,[†] and Gábor Csányi[**,¶]

[†] *Yusuf Hamied Department of Chemistry, University of Cambridge, Lensfield Road, Cambridge, CB2 1EW, UK*

[‡] *School of Natural and Environmental Science, Newcastle University, Newcastle upon Tyne, NE1 7RU, UK*

[¶] *Engineering Laboratory, University of Cambridge, Trumpington St and JJ Thomson Ave, Cambridge, CB2 1PZ, UK*

E-mail: gc121@cam.ac.uk

S1 Details of MLIP training protocols

S1.1 DeePMD

DeePMD models were trained using DeePMD-kit version 2.0.3.[1]

Atomic environments were analysed using the "se_e2_a" descriptor function,[2] also called Deep Potential Smooth Edition (DeepPot-SE). This is a smooth descriptor function that uses an embedding network to learn representative many-body features from an input matrix

consisting of two-atom radial and orientational information. This matrix used a cutoff radius of 6Å, to match the cutoff used in the reference GAP model,[3] and applied a smooth cutoff beginning from 5.5Å. The embedding network had three layers containing 25, 50 and 100 neurons respectively.

The fitting network used to map the descriptor function onto atomic energies and forces comprised three layers with 200 neurons each. The loss function was kept as close as possible to that used in the GAP study: prefactors for the energy, forces, and virials respectively were 1562.5, 48000, and 562.5, which were not varied during the model optimisation. Training consisted of 200,000 steps of the Adam stochastic gradient descent optimiser[4] with a batch size of 5, and a learning rate which decreased gradually from 0.01 at the first step to 1.0×10^{-5} at the final step. We observed that this number of steps was consistently sufficient to achieve a plateau in the loss function, typically with energy errors less than 10 meV (0.1-1 meV/atom) and RMS force errors in the range 10-100 meV/Å depending on the specific configuration. A committee of 5 equivalent models was obtained by varying the random seeds that initialised the networks.

We acknowledge that these parameters have not been carefully optimised and that better performance with DeePMD could be obtained after a careful hyperparameter scan. The intention of this study was to examine the effect of training dataset with consistent hyperparameters, and to establish whether DeePMD models could reproduce the behaviour of GAP models trained using very similar settings.

S1.2 MACE

All models in this paper were trained using MACE version 0.3.6. We employed the standard MACE model architecture[5] with 2 message-passing layers using 128 equivariant messages.

MACE constructs its descriptor functions by combining atomic feature vectors and interatomic displacement vectors expressed in a spherical harmonic basis. The radial part of this basis used Bessel functions up to the 5th polynomial, and a radial cutoff of 6Å to match the

DeePMD and GAP models - however the two message-passing layers gives the MLIP a receptive field radius of 12Å. The spherical harmonic features used an angular cutoff of $l_{\max} = 3$. MACE incorporates many-body information into its descriptors through the tensor-sketching and Clebsch-Gordan contraction procedures. We selected basis functions that contain up to 4-body information (the "correlation" parameter of the MACE training program). However, the message-passing step communicates additional information between atoms, giving the MLIP a total body-order of 13, which may account for the improved performance of this model compared with the simpler approaches.

Training was performed using the AMSGrad variant of the Adam stochastic gradient optimiser with a batch size of 10 and default parameters $\beta_1 = 0.9$ $\beta_2 = 0.999$, and $\epsilon = 1 \times 10^{-8}$. We used the exponential moving average approach to updating weights, with a decay/momentum parameter of 0.99. We allowed a maximum of 750 training epochs but exited early if 50 epochs passed without decreasing the prediction error for a validation set (comprising a randomly-selected 5% of the training data). In practice, we found that this condition was rarely met.

The loss function gave equal weight to energy and virial terms, with a weight 100 times larger on the forces. While these terms are quantitatively different to those used in GAP and DeePMD, they have a similar order of magnitude. We employed the SWA weighting scheme,[6] meaning that the weight of the energy term was increased to 10 times the force weight and the learning rate decreased to 0.001 for the last 250 epochs. In line with previous work in our groups, we found that this approach reduces the RMS error in predicted energies by a factor of 2-4, without significantly worsening the predicted forces.

The final configuration energies and forces were predicted by a readout multilayer perceptron comprised of three 64-node layers, taking the many-body descriptor functions as its input.

S2 MLIP Performance Comparison

Our MLIPs were trained on the University of Cambridge Research Computing Service HPC facility, the Center for Data Driven Discovery (CSD3). DeePMD models were trained and tested using Intel Ice Lake nodes, MACE models using Nvidia Ampere GPUs. We compare the training and inference costs of these two models using the nominal costs provided by the RCS: £0.01/CPUh for Ice Lake and £0.55/GPUh for Ampere.

For DeePMD models, the training time varied quite significantly for different training set sizes and different parallelisation schemes. Training on a single thread, low costs could be achieved (each training step taking an average 0.4 core-seconds or £1×10^{-6} per atomic environment; the total training cost was £0.0002 per environment) at the cost of walltimes running into multiple days. Training on more cores (up to a full 72-core node) cost around £0.00002 per step per environment, and up to £0.1 per environment for a full training run, but reduced the walltime to 10-12 hours. Note that the number of atoms varied considerably between batches, preventing simple comparison with the MACE training time.

MACE model training times were more consistent, and we experimented less with parallelisation parameters. Each training step required approximately 0.4ms per atomic environment, costing £0.0002, the total cost to train was around £0.16 per environment with a walltime of 3-6 hours depending on training set size.

All simulation tests were run on a standard system size of 640 atoms. DeePMD models used a single IceLake node, parallelised across 72 cores using the lammps MPI interface. We achieved approximately 2.5ns/day at a nominal cost of £7 per ns. MACE models were tested on a single Ampere GPU, yielding an average 0.57ns/day of trajectory at a cost of £23.21 per ns.

S3 DeePMD Active Learning protocol

Sec. 3.2 details how transferring greater portions of the GAP training set affects the amount of active learning required to achieve high MLIP performance for DeePMD models. We used the following simple active learning procedure representative of standard practise in the field, in which an initial model generates new configurations and committee disagreement is used to identify which are worst-represented in the original training data. These configurations are labelled with the correct DFT energies (necessitating small system sizes) and then added to the dataset for the next iteration of training. Since we operate in the low-data regime and some trajectories are unstable, we add further criteria to avoid selecting drastically unphysical configurations (e.g. with broken molecules) that we find provide little benefit to model performance. This approach is similar to the "upper trust bound" on the committee disagreement that is sometimes used to exclude failed configurations from an active learning procedure.[7]

The protocol used is as follows:

1. Train a committee of 5 models on the current dataset.One is designated as the primary model. We chose to restart training from new randomly-assigned network weights at each iteration of active learning, to avoid trapping our models in local minima of the optimisation landscape.

2. 25 NPT-ensemble trajectories are obtained using the primary model with a small simulation cell size (12 molecules). We simulate 5 different compositions (EC content varying between 17% and 83%) each with 5 different initial configurations that were originally obtained from a GAP MD trajectory in ref.[3] Each trajectory is run for 500ps, stopping early if any intramolecular bond length exceeds its average value by more than 0.5Å, or if the liquid density leaves the range $[0.2\text{g/cm}^3, 2.0\text{g/cm}^3]$.

3. Select the first configuration from each trajectory that has any atomic force component with a committee disagreement greater than 0.5 $\text{eV}^2/\text{Å}^2$. This disagreement is the

variance in the predictions of all committee members for a given force component, and the threshold corresponds to 10 times the typical RMS force error of 10meV/Å.

4. If no high-disagreement configurations were encountered in 500ps of simulation (or before the early-stopping criteria were hit), we select the configuration with the largest maximum committee disagreement.

5. Recalculate energies of the 25 selected configurations with the PBE-D2 functional using CASTEP, and add them to the training set for the next generation.

Note that the early-stopping conditions stated in step 2 correspond to the definition of trajectory stability described in sec. 2.2. They ensure that we select training configurations that are near but not beyond the boundaries of the physically-accessible configuration space (e.g.. have slightly-stretched bonds and low-probability densities, rather containing broken molecules or evaporating).

S4 Validating diffusion coefficients against reference DFT

Sec. 3.1 showed a moderate systematic difference in centre-of-mass diffusivity between MACE models and the GAP/DeePMD models. We wish to understand which MLIP is closer to the "correct" (DFT) value, but evaluating reference diffusion coefficients using AIMD would be prohibitively expensive. Instead, we study the predictive accuracy of our MLIPs for configurations extracted from canonical trajectories of the models in question, validating the energies and forces of these configurations against the reference DFT method. This approach does not compare predictions for equivalent configurations of the two models, but rather assesses how accurately the MLIP describes the configurations that contribute to its diffusivity calculation. It is also intended to measure how far each model strays from its well-trained configuration space.

We compare two representative MLIPs: a MACE model trained on the Full dataset (which predicts a diffusivity around 15% higher than GAP), and a DeePMD model trained

on the Full dataset plus one generation of active learning. This latter model has a diffusivity very similar to GAP. We sampled configurations at 20ps intervals from a 1ns constant-volume trajectory at a density of 0.92g/cm^3, which is close to the equilibrium density for all three models.

Fig. S1 shows the prediction errors for total energies and force components with each model. The top panels show that the MACE errors remain small and approximately constant through the trajectory: the prediction error is $\approx$ 4meV/atom for the total energy and 25meV/Å for force components. These values are comparable to the equivalent errors reported during model training for the held-out validation set, while the DeePMD errors are several times larger and increase substantially over the first 100ps of simulation. This increase shows that the DeePMD trajectories deviate from the well-trained regions of their parent models and sample configurations that are dissimilar to the training data, likely decreasing the accuracy of their predicted properties. Diffusivity is related to the velocity time-autocorrelation function, so time-dependent changes in the configuration space being explored will have a particularly large effect on the predicted diffusion coefficient. We therefore expect that the MACE diffusivities are closer to the DFT value than this DeePMD model.

The lower left panel of fig. S1 shows that the DeePMD trajectory explores configurations with higher DFT energies than the MACE model, and that these configurations have negative prediction errors (explaining why the model considers them to be easily accessible). Note that this plot does not imply that DeePMD systematically under-predicts the energies of all high-energy configurations (since there may be unexplored configurations for which the converse is true) but it does show that the energy barriers being sampled in this trajectory have a much larger error than the barriers being sampled by the MACE trajectories. Diffusivity will be particularly sensitive to those errors.

The lower right panel shows how the error in a predicted force component correlates with the DFT value of that component. Again, MACE errors are smaller than DeePMD errors

and roughly uncorrelated. The DeePMD data show two populations of force components: there exist a broad spread of atomic environments with comparatively small prediction errors, and a smaller population that have much larger errors (comparable with the magnitude of the DFT force component). This division agrees with the literature explanation that atomic configurations that are very different far from the training set will experience high prediction errors while well-trained configurations do not. The plot also demonstrates that the DeePMD trajectory encounters many such configurations, while the MACE model does not.

We conclude that the MACE models perform more reliably and suffer smaller random errors than DeePMD, and are therefore probably more accurate than GAP as well.

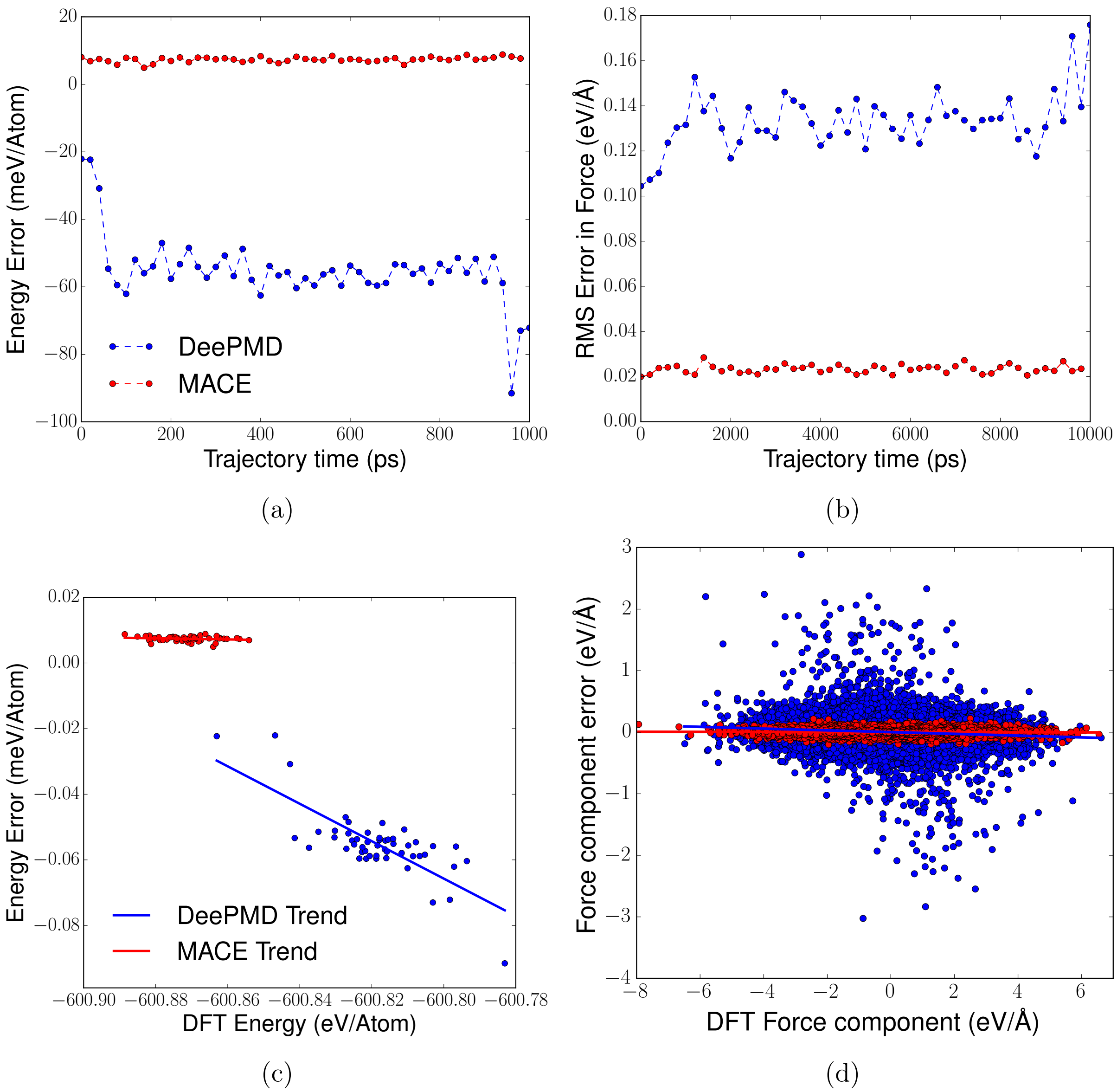

Figure S1: Prediction errors for energies (left) and forces (right) in NVT trajectories with comparable DeePMD and MACE models. a) and b) show how the prediction errors change over time during the trajectory, c) and d) show how the errors correlate with the value of the reference DFT value for each quantity.

References

(1) Wang, H.; Zhang, L.; Han, J.; E, W. DeePMD-kit: A deep learning package for many-body potential energy representation and molecular dynamics. *Computer Physics Communications* **2018**, *228*, 178–184.

(2) Zhang, L.; Han, J.; Wang, H.; Saidi, W.; Car, R.; E, W. End-to-end Symmetry Preserving Inter-atomic Potential Energy Model for Finite and Extended Systems. Advances in Neural Information Processing Systems. 2018.

(3) Magdău, I.-B.; Arismendi-Arrieta, D. J.; Smith, H. E.; Grey, C. P.; Hermansson, K.; Csányi, G. Machine learning force fields for molecular liquids: Ethylene Carbonate/Ethyl Methyl Carbonate binary solvent. *npj Computational Materials* **2023**, *9*, 146.

(4) Kingma, D.; Ba, J. Adam: A Method for Stochastic Optimization. International Conference on Learning Representations (ICLR). San Diego, CA, USA, 2015.

(5) Kovács, D. P.; Batatia, I.; Arany, E. S.; Csányi, G. Evaluation of the MACE force field architecture: From medicinal chemistry to materials science. *The Journal of Chemical Physics* **2023**, *159*, 044118.

(6) Izmailov, P.; Podoprikhin, D.; Garipov, T.; Vetrov, D.; Wilson, A. G. Averaging Weights Leads to Wider Optima and Better Generalization. Uncertainty in Artificial Intelligence. Monterey, CA, USA, 2018.

(7) Hu, J.-Y.; Zhuang, Y.-B.; Cheng, J. Band alignment of CoO(100)–water and CoO(111)–water interfaces accelerated by machine learning potentials. *The Journal of Chemical Physics* **2024**, *161*, 134110.
